# CHARACTERISTICS OF CAREGIVERS WITH FAMILY CONFLICT

**DOI:** 10.1093/geroni/igz038.1809

**Published:** 2019-11-08

**Authors:** JoAnna Dieker, Kelsey Bacharz, Kendall Weber, Sara H Qualls

**Affiliations:** University of Colorado Colorado Springs, Colorado Springs, Colorado, United States

## Abstract

The family environment is often overlooked in caregiver research and assessment, despite having implications for caregiver health and well-being (Zarit et al., 2019). The purpose of the present study was to examine differences on two types of family conflict (beliefs and support) among a diverse sample of caregivers. The present sample consisted of help-seeking (n = 375) and non-help-seeking (n = 415) caregivers (total n = 790). Caregivers filled out the Caregiver Reaction Scale (O’Malley & Qualls, 2017), a multidimensional assessment of the caregiver experience. Results of a 2 (adult children, spouse) x 2 (help-seeking, non-help-seeking) ANOVA indicated that help-seeking caregivers reported significantly more conflict over family beliefs than did non-help-seeking caregivers (M = 1.93 and 1.58, respectively), F(3,606) = 21.10 p < .001. Adult children caregivers reported significantly greater conflict over family beliefs (M = 1.91) than did spouse caregivers (M = 1.60), F(3,606) = 10.66, p < .001. Adult children caregivers also reported significantly greater conflict over family support (M = 1.87) than did spouse caregivers (M = 1.57), F(3,600) = 16.23, p < .001. Results highlight that certain caregiving contexts (e.g., adult children caring for a parent) potentially increase family conflict, which has implications for caregiver burden. Family conflict over beliefs is also related to help-seeking in caregivers. Findings inform appropriate assessment and intervention regarding the family environment in caregiving.

